# Information Geometric Approach on Most Informative Boolean Function Conjecture

**DOI:** 10.3390/e20090688

**Published:** 2018-09-10

**Authors:** Albert No

**Affiliations:** Department of Electronical and Electrical Engineering, Hongik University, Seoul 04066, Korea; albertno@hongik.ac.kr; Tel.: +82-2-320-1649

**Keywords:** Boolean function, Bregman divergence, clustering, geometric mean, Jensen–Shannon divergence

## Abstract

Let $X^{n}$ be a memoryless uniform Bernoulli source and $Y^{n}$ be the output of it through a binary symmetric channel. Courtade and Kumar conjectured that the Boolean function $f:{\left\{0,1\right\}}^{n}\to \left\{0,1\right\}$ that maximizes the mutual information $I(f\left(X^{n}\right);Y^{n})$ is a dictator function, i.e., $f\left(x^{n}\right)=x_{i}$ for some *i*. We propose a clustering problem, which is equivalent to the above problem where we emphasize an information geometry aspect of the equivalent problem. Moreover, we define a normalized geometric mean of measures and interesting properties of it. We also show that the conjecture is true when the arithmetic and geometric mean coincide in a specific set of measures.

## 1. Introduction

Let $X^{n}$ be an independent and identically distributed (i.i.d.) uniform Bernoulli source and $Y^{n}$ be an output of it through a memoryless binary symmetric channel with crossover probability p<1/2. Recently, Courtade and Kumar conjectured that the most informative Boolean function is a dictator function.

**Conjecture** **1** ([1])**.** *For any Boolean function* $f:{\left\{0,1\right\}}^{n}\to \left\{0,1\right\}$*, we have:*

(1) $$\begin{array}{c} I\left(f\left(X^{n}\right);Y^{n}\right)\leq 1-h_{2}\left(p\right) \end{array}$$

*where the maximum is achieved by a dictator function, i.e.,* $f\left(x^{n}\right)=x_{i}$ *for some* 1≤i≤n. *Note that* $h_{2}\left(p\right)=-p\log p-\left(1-p\right)\log\left(1-p\right)$ *is the binary entropy function.*

Although there has been some progress in this line of work [2,3], this simple conjecture still remains open. There are also a number of variations of this conjecture. Weinberger and Shayevitz [4] considered the optimal Boolean function under quadratic loss. Huleihel and Ordentlich [5] considered the complementary case and showed that $I\left(f\left(X^{n}\right);Y^{n}\right)\leq \left(n-1\right)\left(1-h_{2}\left(p\right)\right)$ for all $f:{\left\{0,1\right\}}^{n}\to {\left\{0,1\right\}}^{n-1}$. Nazer et al. focused on information distilling quantizers [6], which can be seen as a generalized version of the above problem.

Many of them are based on the Fourier analysis technique including the original paper [1]. In this paper, we suggest an alternative approach, namely the information geometric approach. The mutual information can naturally be expressed with Kullback–Leibler (KL) divergences. Thus, it can be shown that the maximizing mutual information is equivalent to clustering probability measures under KL divergence.

In the equivalent clustering problem, the center of the cluster is an arithmetic mean of measures. We also provide the role of the geometric mean of measures (with appropriate normalization) in this setting. To the best of our knowledge, the geometric mean of measures has received less attention in the literature. We propose an equivalent formulation of the conjecture using the geometric mean of measures. Note that the geometric mean also allows us to connect Conjecture 1 to the other well-known clustering problem.

The rest of the paper is organized as follows. In Section 2, we briefly review the Jensen–Shannon divergence and I-compressedness. In Section 3, we provide an equivalent clustering problem of probability measures. We introduce the geometric mean of measures in Section 4. We conclude this paper in Section 5.

### Notations

X denotes the alphabet set of random variable *X*, and M(X) denotes the set of measures on X. $X^{n}$ denotes a random vector $\left(X_{1},\;X_{2},\;\ldots ,\;X_{n}\right)$, while $x^{n}$ denotes a specific realization of it. If it is clear from the context, $P_{Y|x}$ denotes a conditional distribution of *Y* given X=x, i.e., $P_{Y|x}\left(y\right)=P_{Y|X}\left(y|x\right)$. Similarly, $P_{Y^{n}|x^{n}}$ denotes a conditional distribution of $Y^{n}$ given $X^{n}=x^{n}$, i.e., $P_{Y^{n}|x^{n}}\left(y^{n}\right)=P_{Y^{n}|X^{n}}\left(y^{n}|x^{n}\right)$. Let $\Omega ={\left\{0,1\right\}}^{n}$ be the set of all binary sequences of length *n*. For A⊆Ω, the shifted version of *A* is denoted by $A⊕x^{n}=\left\{{\tilde{x}}^{n}⊕x^{n}:{\tilde{x}}^{n}\in A\right\}$ where ⊕ is an element-wise XOR operator. The arithmetic mean of measures in the set $\left\{P_{Y^{n}|x^{n}}:x^{n}\in A\right\}$ is denoted by $\mu_{A}$. For 1≤i≤n, let $A_{i0}$ be the set of elements in *A* that satisfy $x_{i}=0$, i.e., $A_{i0}=\left\{x^{n}\in A:x_{i}=0\right\}$, and $\Omega_{i0}=\left\{x^{n}\in \Omega :x_{i}=0\right\}$. $A_{i1}$ is defined in a similar manner. A length *n* binary vector $x^{n-1}0$ denotes a vector $x^{n}$ with $x_{n}=0$.

## 2. Preliminaries

### 2.1. Jensen–Shannon Divergence

For $\alpha_{1},\alpha_{2}\geq 0$ such that $\alpha_{1}+\alpha_{2}=1$, the Jensen–Shannon (JS) divergence of two measures $P_{1}$ and $P_{2}$ is defined as:

(2) $$\begin{array}{c} {\mathrm{JSD}}_{\alpha }\left(P_{1},P_{2}\right)=H\left(\alpha_{1}P_{1}+\alpha_{2}P_{2}\right)-\alpha_{1}H\left(P_{1}\right)-\alpha_{2}H\left(P_{2}\right). \end{array}$$

It is not hard to show that the following definition is equivalent.

(3) $$\begin{array}{c} {\mathrm{JSD}}_{\alpha }\left(P_{1},P_{2}\right)=\alpha_{1}D\left(P_{1}∥\alpha_{1}P_{1}+\alpha_{2}P_{2}\right)+\alpha_{2}D\left(P_{2}∥\alpha_{1}P_{1}+\alpha_{2}P_{2}\right). \end{array}$$

Lin proposed a generalized JS divergence [7]:

(4) $$\begin{array}{c} {\mathrm{JSD}}_{\alpha }\left(P_{1},P_{2},\ldots ,P_{n}\right)=H\left(\sum_{i=1}^{n}\alpha_{i}P_{i}\right)-\sum_{i=1}^{n}\alpha_{i}H\left(P_{i}\right) \end{array}$$

where $\alpha =(\alpha_{1},\ldots ,\alpha_{n})$ is a weight vector such that $\sum_{i=1}^{n}\alpha_{i}=1$. Similar to Equation (3), it has an equivalent definition:

(5) $$\begin{array}{c} {\mathrm{JSD}}_{\alpha }\left(P_{1},\;P_{2},\;\ldots ,\;P_{n}\right)=\sum_{i=1}^{n}\alpha_{i}D\left(P_{i}∥\bar{P}\right) \end{array}$$

where $\bar{P}=\sum_{i=1}^{n}\alpha_{i}P_{i}$. Topsøe [8] pointed out an interesting property, the so-called compensation identity. It states that for any distribution *Q*,

(6) $$\begin{array}{cc} \sum_{i=1}^{n}\alpha_{i}D\left(P_{i}∥Q\right)= & \sum_{i=1}^{n}\alpha_{i}D\left(P_{i}∥\bar{P}\right)+D\left(\bar{P}∥Q\right) \end{array}$$

(7) $$\begin{array}{cc} = & {\mathrm{JSD}}_{\alpha }\left(P_{1},\;P_{2},\;\ldots ,\;P_{n}\right)+D\left(\bar{P}∥Q\right). \end{array}$$

Throughout the paper, we often use Equation (6) directly without the notion of JSD

**Remark** **1.** *The generalized JS divergence is the mutual information between X and the mixture distribution. Let Z be a random variable that takes the value from {1,2,…,n} where $P_{Z}\left(i\right)=\alpha_{i}$ and $P_{X|Z}\left(x|i\right)=P_{i}\left(x\right)$. Then, it is not hard to show that:*

(8) $$\begin{array}{c} {JSD}_{\alpha }\left(P_{1},\;P_{2},\;\ldots ,\;P_{n}\right)=I\left(X;Z\right) \end{array}$$

*However, we introduced generalized JS divergence to emphasize the information geometric perspective of our problem.*

### 2.2. I-Compressed

Let *A* be the subset of Ω and $I=\left\{i_{1},\;i_{2},\;\ldots ,\;i_{k}\right\}\subset \left\{1,\;2,\;\ldots ,\;n\right\}$ be the set of indexes. For $x^{n}\in \Omega$, the I-section of *A* is defined as:

(9) $$\begin{array}{c} A_{I}\left(x^{n}\right)=\left\{z^{k}:y^{n}\in A,y_{i}=\left\{\begin{array}{cc} z_{j} & \mathrm{if}\;i=i_{j}\in I\\ x_{i} & \mathrm{otherwise} \end{array}\right.\right\}. \end{array}$$

The set *A* is called I-compressed if $A_{I}\left(x^{n}\right)$ is an initial segment of lexicographical ordering for all $x^{n}$. For example, if *A* is I-compressed for some |I|=2, then $A_{I}\left(x^{n}\right)$ should be one of:

(10) $$\begin{array}{c} \{00\},\{00,01\},\{00,01,10\},\{00,01,10,11\}. \end{array}$$

It simply says that if $x^{n-2}10\in A$, then $x^{n-2}00,x^{n-2}01\in A$.

Courtade and Kumar showed that it is enough to consider the I-compressed sets.

**Theorem** **1** ([1])**.** *Let $S_{n}$ be the set of functions f:Ω→{0,1} for which $f^{-1}\left(0\right)$ is I-compressed for all I with |I|≤2. In maximizing $I(f\left(X^{n}\right);Y^{n})$, it is sufficient to consider functions $f\in S_{n}$.*

In this paper, we often restrict our attention to functions in the set $S_{n}$.

## 3. Approach via Clustering

In this section, we provide an interesting approach toward Conjecture 1 via clustering. More precisely, we formulate an equivalent clustering problem.

### 3.1. Equivalence to Clustering

The following theorem implies the relation between the original conjecture and the clustering problem.

**Theorem** **2.** *Let f:X→U and U=f(X) be an induced random variable. Then,*

(11) $$\begin{array}{cc} I(f(X);Y)= & I\left(X;Y\right)-\sum_{x}P_{X}\left(x\right)D\left(P_{Y|x}∥P_{Y|U}\left(\cdot |f\left(x\right)\right)\right). \end{array}$$

The proof of the theorem is provided in Appendix A. Note that:

(12) $$\begin{array}{cc} P_{Y|U}\left(y|u\right)= & \frac{P_{U|Y}\left(u|y\right)P_{Y}\left(y\right)}{P_{U}\left(u\right)} \end{array}$$

(13) $$\begin{array}{cc} = & \frac{P_{Y}\left(y\right)}{P_{U}\left(u\right)}\sum_{x\in f^{-1}\left(u\right)}P_{X|Y}\left(x|y\right) \end{array}$$

(14) $$\begin{array}{cc} = & \sum_{x\in f^{-1}\left(u\right)}\frac{P_{X}\left(x\right)}{P_{U}\left(u\right)}\cdot P_{Y|X}\left(y|x\right). \end{array}$$

which is a weighted mean of $P_{Y|X}\left(y|x\right)$ for $x\in f^{-1}\left(u\right)$. The $D(P_{Y|x}∥P_{Y|U}\left(\cdot |f\left(x\right)\right))$ is a distance from each element to the cluster center. This implies that maximizing I(f(X);Y) is equivalent to clustering $\left\{P_{Y^{n}|x^{n}}\right\}$ under KL divergences. Since KL divergence is a Bregman divergence, all clusters are separated by a hyperplane [9].

In this paper, we focus on U={0,1} where $X^{n}$ is i.i.d. Bern(1/2).

**Corollary** **1.** *Let f:Ω→{0,1} and $U=f(X^{n})$ be a binary random variable.*

(15) $$\begin{array}{cc} I(f\left(X^{n}\right);Y^{n})= & n-nh_{2}\left(p\right)-\frac{1}{2^{n}}\sum_{x^{n}}D\left(P_{Y^{n}|x^{n}}∥P_{Y^{n}|U}\left(\cdot |f\left(x^{n}\right)\right)\right). \end{array}$$

The equivalent clustering problem is minimizing:

(16) $$\begin{array}{c} \sum_{x^{n}}D\left(P_{Y^{n}|x^{n}}∥P_{Y^{n}|U}\left(\cdot |f\left(x^{n}\right)\right)\right). \end{array}$$

Let $A=\{x^{n}\in \Omega :f\left(x^{n}\right)=0\}$, then we can simplify $P_{Y^{n}|U}$ further.

(17) $$\begin{array}{cc} P_{Y^{n}|U}\left(y^{n}|0\right)= & \frac{1}{\left|A\right|}\sum_{x^{n}\in A}P_{Y^{n}|X^{n}}\left(y^{n}|x^{n}\right) \end{array}$$

(18) $$\begin{array}{cc} \overset{\Delta }{=} & \mu_{A}\left(y^{n}\right). \end{array}$$

The cluster center $\mu_{A}$ is an arithmetic mean of measures in the set $\left\{P_{Y^{n}|x^{n}}:x^{n}\in A\right\}$. Then, we have:

(19) $$\begin{array}{cc} \sum_{x^{n}}D\left(P_{Y^{n}|x^{n}}∥P_{Y^{n}|U}\left(\cdot |f\left(x^{n}\right)\right)\right)= & \sum_{x^{n}\in A}D\left(P_{Y^{n}|x^{n}}∥\mu_{A}\right)+\sum_{x^{n}\in A^{c}}D\left(P_{Y^{n}|x^{n}}∥\mu_{A^{c}}\right). \end{array}$$

For simplicity, let:

(20) $$\begin{array}{c} D\left(A\right)\overset{\Delta }{=}\sum_{x^{n}\in A}D(P_{Y^{n}|x^{n}}\left|\right|\mu_{A}) \end{array}$$

which is the sum of distances from each element in *A* to the cluster center. In short, finding the most informative Boolean function *f* is equivalent to finding the set A⊆Ω that minimizes $D\left(A\right)+D\left(A^{c}\right)$.

**Remark** **2.** *Conjecture 1 implies that $A=\Omega_{i0}=\left\{x^{n}:x_{i}=0\right\}$ minimizes (19). Furthermore, Theorem 1 implies that it is enough to consider A such that $A_{i1}\subseteq A_{i0}$ for all i.*

For any $Q_{Y^{n}}\in M\left(\Omega \right)$, Equation (6) implies that:

(21) $$\begin{array}{c} \sum_{x\in A}D\left(P_{Y^{n}|x^{n}}∥Q_{Y^{n}}\right)=D\left(A\right)+\left|A\right|D\left(\mu_{A}∥Q_{Y^{n}}\right) \end{array}$$

(22) $$\begin{array}{c} \sum_{x\in A^{c}}D\left(P_{Y^{n}|x^{n}}∥Q_{Y^{n}}\right)=D\left(A^{c}\right)+\left|A^{c}\right|D\left(\mu_{A^{c}}∥Q_{Y^{n}}\right). \end{array}$$

Thus, we have:

(23) $$\begin{array}{c} \sum_{x\in \Omega }D\left(P_{Y^{n}|x^{n}}∥Q_{Y^{n}}\right)=D\left(A\right)+D\left(A^{c}\right)+|A|D\left(\mu_{A}∥Q_{Y^{n}}\right)+\left|A^{c}\right|D\left(\mu_{A^{c}}∥Q_{Y^{n}}\right). \end{array}$$

Note that $\sum_{x\in \Omega }D\left(P_{Y^{n}|x^{n}}∥Q_{Y^{n}}\right)$ does not depend on *A*, and therefore, we have the following theorem.

**Theorem** **3.** *For any $Q_{Y^{n}}\in M\left(\Omega \right)$, minimizing $D\left(A\right)+D\left(A^{c}\right)$ is equivalent to maximizing:*

(24) $$\begin{array}{c} |A|D\left(\mu_{A}∥Q_{Y^{n}}\right)+\left|A^{c}\right|D\left(\mu_{A^{c}}∥Q_{Y^{n}}\right). \end{array}$$

The above theorem provides an alternative problem formulation of the original conjecture.

### 3.2. Connection to Clustering under Hamming Distance

In this section, we consider the duality between the above clustering problem under the KL divergence and the clustering on Ω under the Hamming distance. The following theorem shows that the KL divergence on $\left\{P_{Y^{n}|x^{n}}:x^{n}\in \Omega \right\}$ corresponds to the Hamming distance on Ω.

**Theorem** **4.** *For all $x^{n},{\tilde{x}}^{n}\in \Omega$, we have:*

(25) $$\begin{array}{c} D\left(P_{Y^{n}|x^{n}}∥P_{Y^{n}{|}^{{\tilde{x}}^{n}}}\right)=d_{H}\left(x^{n},{\tilde{x}}^{n}\right)\cdot \left(1-2p\right)\log\frac{1-p}{p} \end{array}$$

*where $d_{H}\left(x^{n},{\tilde{x}}^{n}\right)$ denotes the Hamming distance between $x^{n}$ and ${\tilde{x}}^{n}$.*

This theorem implies that the distance between two measures $P_{Y^{n}|x^{n}}$ and $P_{Y^{n}|{\tilde{x}}^{n}}$ is proportional to the Hamming distance between two binary vectors $x^{n}$ and ${\tilde{x}}^{n}$. The proof of the theorem is provided in Appendix B. Note that the KL divergence D(·∥·) is symmetric on $\left\{P_{Y^{n}|x^{n}}:x^{n}\in \Omega \right\}$.

In the above duality, we have a mapping between $\left\{P_{Y^{n}|x^{n}}:x^{n}\in \Omega \right\}$ and ${\left\{0,1\right\}}^{n}$; more precisely, $P_{Y^{n}|x^{n}}↔x^{n}$. This mapping naturally suggests an equivalent clustering problem of *n*-dimensional binary vectors. However, the cluster center $\mu_{A}$ is not an element of $\left\{P_{Y^{n}|x^{n}}:x^{n}\in \Omega \right\}$ in general. In order to formulate an equivalent clustering problem, we need to answer the question “Which *n* dimensional vector corresponds to $\mu_{A}$?”. A naive approach is to extend the set of binary vectors to ${\left[0,1\right]}^{n}$ under $\ell^{2}$ distance instead of the Hamming distance. In such a case, the goal is to map $\mu_{A}$ to the arithmetic mean of binary vectors in the set *A*. If this is true, we can further simplify the problem into the problem of clustering a hypercube in $R^{n}$. However, the following example shows that this naive extension is not valid.

**Example** **1.** *Let n=2, A={00,11} and B={01,10}, then the arithmetic mean of binary vectors of A and that of B are the same. However, $\mu_{A}$ is not equal to $\mu_{B}$.*

Furthermore, the set $\Omega_{i0}$ is not the optimum choice when clustering the hypercube under $\ell^{2}$. Instead, we need to consider the set of measures directly. The following theorem provides a bit of geometric structure among measures.

**Theorem** **5.** *For all $x^{n},{\tilde{x}}^{n}\in \Omega$ and $Q_{Y^{n}}\in \left(\left\{P_{Y^{n}|x^{n}}|x^{n}\in \Omega \right\}\right)$,*

(26) $$\begin{array}{cc} D\left(P_{Y^{n}|X^{n}=x^{n}}∥Q_{Y^{n}}\right)-D\left(P_{Y^{n}|X^{n}={\tilde{x}}^{n}}∥Q_{Y^{n}}\right)\leq & k\cdot \left({\left(1-p\right)}^{k}-p^{k}\right)\log\frac{1-p}{p} \end{array}$$

*where $k=d_{H}\left(x^{n},{\tilde{x}}^{n}\right)$.*

The proof of the theorem is provided in Appendix C. Since ${\left(1-p\right)}^{k}-p^{k}\leq 1-2p$ for all k≥1, Theorem 5 immediately implies the following corollary.

**Corollary** **2.** *For all $x^{n},{\tilde{x}}^{n}\in \Omega$ and $Q_{Y^{n}}\in \mathrm{conv}\left(\left\{P_{Y^{n}|x^{n}}|x^{n}\in \Omega \right\}\right)$,*

(27) $$\begin{array}{c} \left|D\left(P_{Y^{n}|X^{n}=x^{n}}∥Q_{Y^{n}}\right)-D\left(P_{Y^{n}|X^{n}={\tilde{x}}^{n}}∥Q_{Y^{n}}\right)\right|\leq D\left(P_{Y^{n}|X^{n}=x^{n}}∥P_{Y^{n}|X^{n}={\tilde{x}}^{n}}\right) \end{array}$$

*where conv(A) is a convex hull of measures in the set A.*

This is a triangle inequality that can be useful when we consider the clustering problem of measures.

## 4. Geometric Mean of Measures

In the previous section, we formulate the clustering problem that is equivalent to the original maximizing mutual information problem. In this section, we provide another approach using a geometric mean of measures. We define the geometric mean of measures formally and derive a nontrivial conjecture, which is equivalent to Conjecture 1.

### 4.1. Definition of the Geometric Mean of Measures

For measures $P_{1},\;P_{2},\;\ldots ,\;P_{n}\in M\left(X\right)$ and weights $\alpha_{i}\geq 0$ such that $\sum_{i=1}^{n}\alpha_{i}=1$, we considered the sum of KL divergences in (6):

(28) $$\begin{array}{c} \sum_{i=1}^{n}\alpha_{i}D\left(P_{i}∥Q\right). \end{array}$$

We also observed that (28) is minimized when *Q* is an arithmetic mean of measures.

Since the KL divergence is asymmetric, it is natural to consider the sum of another direction of KL divergences.

(29) $$\begin{array}{cc} \sum_{i=1}^{n}\alpha_{i}D\left(Q∥P_{i}\right)= & \sum_{i=1}^{n}\alpha_{i}\sum_{x\in X}Q\left(x\right)\log\frac{Q(x)}{P_{i}\left(x\right)} \end{array}$$

(30) $$\begin{array}{cc} = & \sum_{x\in X}\sum_{i=1}^{n}\alpha_{i}Q\left(x\right)\log\frac{Q(x)}{P_{i}\left(x\right)} \end{array}$$

(31) $$\begin{array}{cc} = & \sum_{x\in X}Q\left(x\right)\log\frac{Q(x)}{\prod_{i=1}^{n}{\left(P_{i}\left(x\right)\right)}^{\alpha_{i}}}. \end{array}$$

Compared to the arithmetic mean that minimizes (28), $\prod_{i=1}^{n}{\left(P_{i}\left(x\right)\right)}^{\alpha_{i}}$ can be considered as a geometric mean of measures. However, $\prod_{i=1}^{n}{\left(P_{i}\left(x\right)\right)}^{\alpha_{i}}$ is not a measure in general, and normalization is required. With a normalizing constant *s*, we can define the geometric mean of measures by:

(32) $$\begin{array}{c} {\bar{P}}_{G}\left(x\right)=\frac{1}{s}\prod_{i=1}^{n}{\left(P_{i}\left(x\right)\right)}^{\alpha_{i}} \end{array}$$

where *s* is a constant, so that $\sum_{x\in X}{\bar{P}}_{G}\left(x\right)=1$, i.e.,

(33) $$\begin{array}{c} s=\sum_{x}\prod_{i=1}^{n}{\left(P_{i}\left(x\right)\right)}^{\alpha_{i}}. \end{array}$$

Then, we have:

(34) $$\sum_{i=1}^{n}\alpha_{i}D\left(Q∥P_{i}\right)=D\left(Q∥{\bar{P}}_{G}\right)+\log\frac{1}{s}$$

which can be minimized when $Q={\bar{P}}_{G}$. Thus, for all *Q*,

(35) $$\begin{array}{cc} \sum_{i=1}^{n}\alpha_{i}D\left(Q∥P_{i}\right)\geq & \sum_{i=1}^{n}\alpha_{i}D\left({\bar{P}}_{G}∥P_{i}\right) \end{array}$$

(36) $$=\log\frac{1}{s}.$$

The above result provides a geometric compensation identity.

(37) $$\begin{array}{cc} \sum_{i=1}^{n}\alpha_{i}D\left(Q∥P_{i}\right)= & D\left(Q∥{\bar{P}}_{G}\right)+\sum_{i=1}^{n}\alpha_{i}D\left({\bar{P}}_{G}∥P_{i}\right). \end{array}$$

This also implies that $\log\frac{1}{s}\geq 0$.

**Remark** **3.** *If n=2, s is called the α-Chernoff coefficient, and it is called the Bhattacharyya coefficient when α=1/2. The summation $\log\frac{1}{s}=\sum_{i=1}^{2}\alpha_{i}D\left({\bar{P}}_{G}∥P_{i}\right)$ is known as α-Chernoff divergence. For more details, please see [10,11] and the references therein.*

Under this definition, we can find the geometric mean of measures in the set $\left\{P_{Y^{n}|{\tilde{x}}^{n}}:{\tilde{x}}^{n}\in B\right\}$ with uniform weights $\frac{1}{\left|B\right|}$ by:

(38) $$\begin{array}{c} \gamma_{B}\left(y^{n}\right)=\frac{1}{s_{B}}{\left(\prod_{{\tilde{x}}^{n}\in B}P_{Y^{n}|X^{n}}\left(y^{n}|{\tilde{x}}^{n}\right)\right)}^{1/|B|} \end{array}$$

where:

(39) $$\begin{array}{c} s_{B}=\sum_{y^{n}}{\left(\prod_{{\tilde{x}}^{n}\in B}P_{Y^{n}|X^{n}}\left(y^{n}|{\tilde{x}}^{n}\right)\right)}^{1/|B|}. \end{array}$$

**Remark** **4.** *The original conjecture is that the Boolean function f such that $f^{-1}\left(0\right)=\Omega_{i0}=\left\{x^{n}:x_{i}=0\right\}$ maximizes the mutual information $I(f\left(X^{n}\right);Y^{n})$. The geometric mean of measures in the set $\left\{P_{Y^{n}|x^{n}}:x^{n}\in \Omega_{i0}\right\}$ satisfies the following property.*

(40) $$\begin{array}{cc} \gamma_{\Omega_{i0}}= & \mu_{\Omega_{i0}} \end{array}$$

(41) $$\begin{array}{cc} s_{\Omega_{i0}}= & 2^{n-1}{\left(p(1-p)\right)}^{(n-1)/2}. \end{array}$$

*Note that the geometric mean of measures in the set $\left\{P_{Y^{n}|x^{n}}:x^{n}\in \Omega \right\}$ satisfies:*

(42) $$\gamma_{\Omega }=\mu_{\Omega }$$

(43) $$s_{\Omega }=2^{n}{\left(p(1-p)\right)}^{n/2}.$$

### 4.2. Main Results

So far, we have seen two means of measures $\mu_{A}$ and $\gamma_{A}$. It is natural to ask if they are equal. Our main theorem provides a connection to Conjecture 1.

**Theorem** **6.** Suppose A is a nontrivial subset of $\Omega ={\left\{0,1\right\}}^{n}$ for n>0 (i.e., A≠∅,Ω), and A is I-compressed for all |I|=1. Then, $A=\Omega_{i0}$ for some i if and only if $\mu_{A}=\gamma_{A}$ and $\mu_{A^{c}}=\gamma_{A^{c}}$.

The proof of the theorem is provided in Appendix D. Theorem 6 implies that the following conjecture is the equivalent to Conjecture 1.

**Conjecture** **2.** *Let f:Ω→{0,1} and $A=f^{-1}\left(0\right)$ be I-compressed for all |I|=1. Then, I(f(X);Y) is maximized if and only if $\mu_{A}=\gamma_{A}$ and $\mu_{A^{c}}=\gamma_{A^{c}}$*.

**Remark** **5.** One of the main challenges of this problem is that the conjectured optimal sets are extremes, i.e., $A=\Omega_{i0}$ for some i. Our main theorem provides an alternative conjecture that seems more natural in the context of optimization.

**Remark** **6.** It is clear that $\mu_{A}=\gamma_{A}$ holds if |A|=1. Thus, both conditions $\mu_{A}=\gamma_{A}$ and $\mu_{A^{c}}=\gamma_{A^{c}}$ are needed to guarantee $A=\Omega_{i0}$ for some i.

### 4.3. Property of the Geometric Mean

We can derive a new identity by combining the original and geometric compensation identity together. For A,B⊂Ω, let π(A,B) be:

(44) $$\begin{array}{c} \pi \left(A,B\right)=\sum_{(x^{n},{\tilde{x}}^{n})\in A\times B}D\left(P_{Y^{n}|x^{n}}∥P_{Y^{n}|{\tilde{x}}^{n}}\right). \end{array}$$

Then,

(45) $$\begin{array}{cc} \pi (A,B)= & \sum_{(x^{n},{\tilde{x}}^{n})\in A\times B}D\left(P_{Y^{n}|x^{n}}∥P_{Y^{n}|{\tilde{x}}^{n}}\right) \end{array}$$

(46) $$\begin{array}{cc} = & \sum_{{\tilde{x}}^{n}\in B}\sum_{x^{n}\in A}D\left(P_{Y^{n}|x^{n}}∥P_{Y^{n}|{\tilde{x}}^{n}}\right) \end{array}$$

(47) $$\begin{array}{cc} = & \sum_{{\tilde{x}}^{n}\in B}\left(\sum_{x^{n}\in A}D\left(P_{Y^{n}|x^{n}}∥\mu_{A}\right)+\left|A\right|D\left(\mu_{A}∥P_{Y|{\tilde{x}}^{n}}\right)\right) \end{array}$$

(48) $$\begin{array}{cc} = & |B|D\left(A\right)+\left|A\right|\sum_{{\tilde{x}}^{n}\in B}D\left(\mu_{A}∥P_{Y|{\tilde{x}}^{n}}\right) \end{array}$$

where (47) is because of the compensation identity (6). As we discussed in Section 4.1, the second term of the right-hand side is:

(49) $$\begin{array}{cc} \sum_{{\tilde{x}}^{n}\in B}D\left(\mu_{A}∥P_{{\tilde{x}}^{n}}\right)= & \sum_{{\tilde{x}}^{n}\in B}\sum_{y^{n}}\mu_{A}\left(y^{n}\right)\log\frac{\mu_{A}\left(y^{n}\right)}{P_{Y^{n}|X^{n}}\left(y^{n}|{\tilde{x}}^{n}\right)} \end{array}$$

(50) $$\begin{array}{cc} = & \left|B\right|\sum_{y^{n}}\mu_{A}\left(y^{n}\right)\log\frac{\mu_{A}\left(y^{n}\right)}{{\left(\prod_{{\tilde{x}}^{n}\in B}P_{Y^{n}|X^{n}}\left(y^{n}|{\tilde{x}}^{n}\right)\right)}^{1/|B|}} \end{array}$$

(51) $$\begin{array}{cc} = & |B|D\left(\mu_{A}∥\gamma_{B}\right)+\left|B\right|\log\frac{1}{s_{B}} \end{array}$$

(52) $$\begin{array}{cc} = & |B|D\left(\mu_{A}∥\gamma_{B}\right)+\sum_{{\tilde{x}}^{n}\in B}D\left(\gamma_{B}∥P_{Y^{n}|{\tilde{x}}^{n}}\right). \end{array}$$

Finally, we have:

(53) $$\begin{array}{cc} \frac{1}{\left|A||B\right|}\pi \left(A,B\right)= & \frac{1}{\left|A\right|}D\left(A\right)+D\left(\mu_{A}∥\gamma_{B}\right)+\log\frac{1}{s_{B}} \end{array}$$

(54) $$\begin{array}{cc} = & \frac{1}{\left|A\right|}\sum_{x^{n}\in A}D\left(P_{Y^{n}|x^{n}}∥\mu_{A}\right)+D\left(\mu_{A}∥\gamma_{B}\right)+\frac{1}{\left|B\right|}\sum_{{\tilde{x}}^{n}\in B}D\left(\gamma_{B}∥P_{Y^{n}|{\tilde{x}}^{n}}\right). \end{array}$$

More interestingly, we can apply original and geometric compensation identities:

(55) $$\begin{array}{cc} \frac{1}{\left|A||B\right|}\pi \left(A,B\right)= & \frac{1}{\left|A\right|}\sum_{x^{n}\in A}D\left(P_{Y^{n}|x^{n}}∥\mu_{A}\right)+\frac{1}{\left|B\right|}\sum_{{\tilde{x}}^{n}\in B}D\left(\mu_{A}∥P_{Y^{n}|{\tilde{x}}^{n}}\right) \end{array}$$

(56) $$\begin{array}{cc} = & \frac{1}{\left|A\right|}\sum_{x^{n}\in A}D\left(P_{Y^{n}|x^{n}}∥\gamma_{B}\right)+\frac{1}{\left|B\right|}\sum_{{\tilde{x}}^{n}\in B}D\left(\gamma_{B}∥P_{Y^{n}|{\tilde{x}}^{n}}\right). \end{array}$$

From Theorem 4, we have π(A,B)=π(B,A), and therefore, we can switch *A* and *B*.

(57) $$\begin{array}{cc} \frac{1}{\left|A||B\right|}\pi \left(A,B\right)= & \frac{1}{\left|B\right|}\sum_{x^{n}\in B}D\left(P_{Y^{n}|x^{n}}∥\mu_{B}\right)+D\left(\mu_{B}∥\gamma_{A}\right)+\frac{1}{\left|A\right|}\sum_{{\tilde{x}}^{n}\in A}D\left(\gamma_{A}∥P_{Y^{n}|{\tilde{x}}^{n}}\right) \end{array}$$

(58) $$\begin{array}{cc} = & \frac{1}{\left|B\right|}\sum_{x^{n}\in B}D\left(P_{Y^{n}|x^{n}}∥\mu_{B}\right)+\frac{1}{\left|A\right|}\sum_{{\tilde{x}}^{n}\in A}D\left(\mu_{B}∥P_{Y^{n}|{\tilde{x}}^{n}}\right) \end{array}$$

(59) $$\begin{array}{cc} = & \frac{1}{\left|B\right|}\sum_{x^{n}\in B}D\left(P_{Y^{n}|x^{n}}∥\gamma_{A}\right)+\frac{1}{\left|A\right|}\sum_{{\tilde{x}}^{n}\in A}D\left(\gamma_{A}∥P_{Y^{n}|{\tilde{x}}^{n}}\right). \end{array}$$

If we let A=B, we have:

(60) $$\begin{array}{cc} \frac{1}{{\left|A\right|}^{2}}\pi \left(A,A\right)= & \frac{1}{\left|A\right|}\sum_{x^{n}\in A}\left[D\left(P_{Y^{n}|x^{n}}∥\gamma_{A}\right)+D\left(\gamma_{A}∥P_{Y^{n}|x^{n}}\right)\right] \end{array}$$

(61) $$\begin{array}{cc} = & \frac{1}{\left|A\right|}\sum_{x^{n}\in A}\left[D\left(P_{Y^{n}|x^{n}}∥\mu_{A}\right)+D\left(\mu_{A}∥P_{Y^{n}|x^{n}}\right)\right] \end{array}$$

(62) $$\begin{array}{cc} = & \frac{1}{\left|A\right|}\sum_{x^{n}\in A}\left[D\left(P_{Y^{n}|x^{n}}∥\mu_{A}\right)+D\left(\mu_{A}∥\gamma_{A}\right)+D\left(\gamma_{A}∥P_{Y^{n}|x^{n}}\right)\right]. \end{array}$$

Note that $\frac{1}{\left|A\right|}\pi \left(A,A\right)+\frac{1}{|A^{c}|}\pi \left(A^{c},A^{c}\right)$ is similar to a known clustering problem. In the clustering literature, the min-sum clustering problem [12] is minimizing the sum of all edges in each cluster. Using π, we can describe the binary min-sum clustering problem on Ω by minimizing $\pi \left(A,A\right)+\pi \left(A^{c},A^{c}\right)$.

### 4.4. Another Application of the Geometric Mean

Using the geometric mean of measures, we can rewrite the clustering problem in a different form. Recall that $\mu_{A⊕x^{n}}=\left\{{\tilde{x}}^{n}⊕x^{n}:{\tilde{x}}^{n}\in A\right\}$. Then, we have:

(63) $$\begin{array}{cc} D(A)= & \sum_{x^{n}\in A}D\left(P_{Y^{n}|x^{n}}∥\mu_{A}\right) \end{array}$$

(64) $$\begin{array}{cc} = & \sum_{x^{n}\in A}D\left(P_{Y^{n}|0^{n}}∥\mu_{A⊕x^{n}}\right). \end{array}$$

Let ${\tilde{\gamma }}_{A}$ be the geometric mean of measures in the set $\left\{\mu_{A⊕x^{n}}:x^{n}\in A\right\}$, i.e.,

(65) $$\begin{array}{c} {\tilde{\gamma }}_{A}\left(y^{n}\right)=\frac{1}{{\tilde{s}}_{A}}{\left(\prod_{x^{n}\in A}\mu_{A⊕x^{n}}\left(y^{n}\right)\right)}^{1/|A|} \end{array}$$

where:

(66) $$\begin{array}{c} {\tilde{s}}_{A}=\sum_{y^{n}}{\left(\prod_{x^{n}\in A}\mu_{A⊕x^{n}}\left(y^{n}\right)\right)}^{1/|A|}. \end{array}$$

Then, we have:

(67) $$\begin{array}{c} D\left(A\right)=|A|D\left(P_{Y^{n}|0^{n}}∥{\tilde{\gamma }}_{A}\right)+\left|A\right|\log\frac{1}{{\tilde{s}}_{A}}. \end{array}$$

Needless to say:

(68) $$\begin{array}{c} D\left(A^{c}\right)=|A^{c}|D\left(P_{Y^{n}|0^{n}}∥{\tilde{\gamma }}_{A^{c}}\right)+\left|A^{c}\right|\log\frac{1}{{\tilde{s}}_{A^{c}}}. \end{array}$$

The sum of the results is:

(69) $$\begin{array}{cc} D\left(A\right)+D\left(A^{c}\right)= & |A|D\left(P_{Y^{n}|0^{n}}∥{\tilde{\gamma }}_{A}\right)+|A^{c}|D\left(P_{Y^{n}|0^{n}}∥{\tilde{\gamma }}_{A^{c}}\right)+|A|\log\frac{1}{{\tilde{s}}_{A}}+\left|A^{c}\right|\log\frac{1}{{\tilde{s}}_{A^{c}}}. \end{array}$$

This can be considered as a dual of Theorem 3.

**Remark** **7.** *Let $\Omega_{i0}=\left\{x^{n}:x_{i}=0\right\}$, which is the candidate of the optimizer. Then,*

(70) $$\begin{array}{cc} {\tilde{\gamma }}_{\Omega_{i0}}= & {\tilde{\gamma }}_{{\left(\Omega_{i0}\right)}^{c}}=\mu_{\Omega_{i0}} \end{array}$$

(71) $$\begin{array}{cc} {\tilde{s}}_{\Omega_{i0}}= & {\tilde{s}}_{{\left(\Omega_{i0}\right)}^{c}}=1. \end{array}$$

## 5. Concluding Remarks

In this paper, we have proposed a number of different formulations of the most informative Boolean function conjecture. Most of them are based on the information geometric approach. Furthermore, we focused on the (normalized) geometric mean of measures that can simplify the problem formulation. More precisely, we showed that Conjecture 1 is true if and only if the maximum achieving *f* satisfies the following property: “the arithmetic and geometric mean of measures are the same for both $\left\{P_{Y^{n}|x^{n}}:x^{n}\in f^{-1}\left(0\right)\right\}$, as well as $\left\{P_{Y^{n}|x^{n}}:x^{n}\in f^{-1}\left(1\right)\right\}$.”

## Appendix A. Proof of Theorem 2

By the definition of mutual information, we have:

(A1) $$\begin{array}{cc} I(X;Y)-I(f(X);Y)= & E\left[\log\frac{P_{X,Y}\left(X,Y\right)}{P_{X}\left(X\right)P_{Y}\left(Y\right)}\right]-E\left[\log\frac{P_{U,Y}\left(f\left(X\right),Y\right)}{P_{U}\left(f\left(X\right)\right)P_{Y}\left(Y\right)}\right] \end{array}$$

(A2) $$\begin{array}{cc} = & E\left[\log\frac{P_{Y|X}\left(Y|X\right)}{P_{Y|U}\left(Y\right|f\left(X\right)}\right] \end{array}$$

(A3) $$\begin{array}{cc} = & E\left[E[\log\frac{P_{Y|X}\left(Y|X\right)}{P_{Y|U}\left(Y|f\left(X\right)\right)}|X]\right] \end{array}$$

(A4) $$\begin{array}{cc} = & \sum_{x}P_{X}\left(x\right)E\left[\log\frac{P_{Y|X}\left(Y|X\right)}{P_{Y|U}\left(Y|f\left(X\right)\right)}|X=x\right] \end{array}$$

(A5) $$\begin{array}{cc} = & \sum_{x}P_{X}\left(x\right)D\left(P_{Y|x}∥P_{Y|U}\left(\cdot |f\left(x\right)\right)\right). \end{array}$$

This concludes the proof.

## Appendix B. Proof of Theorem 4

Without loss of generality, we can assume that $x^{n}=0^{n}$ and ${\tilde{x}}^{n}=1^{k}0^{n-k}$ where $k=d_{H}\left(x^{n},{\tilde{x}}^{n}\right)$. Then, we have:

$$\begin{array}{c} D(P_{Y^{n}|x^{n}}∥P_{Y^{n}|{\tilde{x}}^{n}}) \end{array}$$

(A6) $$\begin{array}{cc} = & D(P_{Y^{k}|x^{k}}\times P_{Y_{k+1}^{n}|x_{k+1}^{n}}∥P_{Y^{k}|{\tilde{x}}^{k}}\times P_{Y_{k+1}^{n}|{\tilde{x}}_{k+1}^{n}}) \end{array}$$

(A7) $$\begin{array}{cc} = & D(P_{Y^{k}|0^{k}}\times P_{Y_{k+1}^{n}|0_{k+1}^{n}}∥P_{Y^{k}|1^{k}}\times P_{Y_{k+1}^{n}|0_{k+1}^{n}}) \end{array}$$

(A8) $$\begin{array}{cc} = & D\left(P_{Y^{k}|0^{k}}∥P_{Y^{k}|1^{k}}\right)+D\left(P_{Y_{k+1}^{n}|0_{k+1}^{n}}∥P_{Y_{k+1}^{n}|0_{k+1}^{n}}\right) \end{array}$$

(A9) $$\begin{array}{cc} = & D(P_{Y^{k}|0^{k}}∥P_{Y^{k}|1^{k}}) \end{array}$$

Thus,

$$\begin{array}{c} D(P_{Y^{k}|0^{k}}∥P_{Y^{k}|1^{k}}) \end{array}$$

(A10) $$\begin{array}{cc} = & kD(P_{Y|0}∥P_{Y|1}) \end{array}$$

(A11) $$\begin{array}{cc} = & k\left(p\log\frac{p}{1-p}+\left(1-p\right)\log\frac{1-p}{p}\right) \end{array}$$

(A12) $$\begin{array}{cc} = & k\left(1-2p\right)\log\frac{1-p}{p}. \end{array}$$

Since $d_{H}\left(x^{n},{\tilde{x}}^{n}\right)=k$, this concludes the proof.

## Appendix C. Proof of Theorem 5

The following lemma bounds the ratio between $Q_{Y}^{n}\left(y^{n}\right)$ and $Q_{Y^{n}}\left({\tilde{y}}^{n}\right)$, which will be crucial in our argument.
**Lemma** **A1.** *For $Q_{Y^{n}}\in \mathrm{conv}\left\{P_{Y^{n}|x^{n}}|x^{n}\in \Omega \right\}$,*

(A13) $$d_{H}\left(y^{n},{\tilde{y}}^{n}\right)\cdot \log\left(\frac{p}{1-p}\right)\leq \log\frac{Q_{Y^{n}}\left(y^{n}\right)}{Q_{Y^{n}}\left({\tilde{y}}^{n}\right)}\leq d_{H}\left(y^{n},{\tilde{y}}^{n}\right)\cdot \log\left(\frac{1-p}{p}\right)$$

**Proof.** Without loss of generality, we can assume that ${\bar{y}}^{k}={\tilde{y}}^{k}$ and $y_{k+1}^{n}={\tilde{y}}_{k+1}^{n}$.

(A14) $$\frac{Q_{Y^{n}}\left(y^{n}\right)}{Q_{Y^{n}}\left({\bar{y}}^{k},y_{k+1}^{n}\right)}=\frac{\sum_{x^{n}}\pi \left(x^{n}\right)P_{Y^{n}|X^{n}}\left(y^{n}|x^{n}\right)}{\sum_{x^{n}}\pi \left(x^{n}\right)P_{Y^{n}|X^{n}}\left({\bar{y}}^{k},y_{k+1}^{n}|x^{n}\right)}$$

(A15) $$\leq {\left(\frac{1-p}{p}\right)}^{k}\frac{\sum_{x^{n}}\pi \left(x^{n}\right)P_{Y^{n}|X^{n}}\left(y^{n}|x^{n}\right)}{\sum_{x^{n}}\pi \left(x^{n}\right)P_{Y^{n}|X^{n}}\left(y^{n}|x^{n}\right)}$$

(A16) $$={\left(\frac{1-p}{p}\right)}^{k}.$$

Similarly, we can show that:

(A17) $$\frac{Q_{Y^{n}}\left(y^{n}\right)}{Q_{Y^{n}}\left({\bar{y}}^{k},y_{k+1}^{n}\right)}\geq {\left(\frac{p}{1-p}\right)}^{k}.$$

This concludes the proof of the lemma. ☐

Without loss of generality, we can assume that $x^{n}=0^{n}$ and ${\tilde{x}}^{n}=1^{k}0^{n-k}$. Then, we have:

(A18) $$P_{Y^{n}|X^{n}}\left(y^{n}|{\tilde{x}}^{n}\right)=P_{Y^{n}|X^{n}}\left(y^{n}|1^{k}0^{n-k}\right)$$

(A19) $$=P_{Y^{n}|X^{n}}\left({\bar{y}}^{k},y_{k+1}^{n}|0^{n}\right)$$

where ${\bar{y}}_{i}=1-y_{i}$. Thus, we have:

$$\begin{array}{c} D\left(P_{Y^{n}|X^{n}=x^{n}}∥Q_{Y^{n}}\right)-D\left(P_{Y^{n}|X^{n}={\tilde{x}}^{n}}∥Q_{Y^{n}}\right) \end{array}$$

(A20) $$\begin{array}{c} =E\left[\log\frac{P_{Y^{n}|X^{n}}\left(Y^{n}|0^{n}\right)}{Q_{Y^{n}}\left(Y^{n}\right)}\right]-E\left[\log\frac{P_{Y^{n}|X^{n}}\left({\bar{Y}}^{k},Y_{k+1}^{n}|0^{n}\right)}{Q_{Y^{n}}\left(Y^{n}\right)}\right] \end{array}$$

(A21) $$=E\left[\log\frac{P_{Y^{n}|X^{n}}\left(Y^{n}|0^{n}\right)}{Q_{Y^{n}}\left(Y^{n}\right)}\right]-E\left[\log\frac{P_{Y^{n}|X^{n}}\left(Y^{n}|0^{n}\right)}{Q_{Y^{n}}\left({\bar{Y}}^{k},Y_{k+1}^{n}\right)}\right]$$

(A22) $$=E\left[\log\frac{Q_{Y^{n}}\left(Y^{n}\right)}{Q_{Y^{n}}\left({\bar{Y}}^{k},Y_{k+1}^{n}\right)}\right].$$

Note that two expectations in (A20) are under different distributions; on the other hand, expectations in (A21) and the following equations are under the same distribution $P_{Y^{n}|X^{n}=0^{n}}$.

The above expectation can be written as follows.

$$\begin{array}{c} D\left(P_{Y^{n}|X^{n}=x^{n}}∥Q_{Y^{n}}\right)-D\left(P_{Y^{n}|X^{n}={\tilde{x}}^{n}}∥Q_{Y^{n}}\right) \end{array}$$

(A23) $$\begin{array}{c} =\sum_{y^{n}}P_{Y^{n}|X^{n}}\left(y^{n}|0^{n}\right)\log\frac{Q_{Y^{n}}\left(y^{n}\right)}{Q_{Y^{n}}\left({\bar{y}}^{k},y_{k+1}^{n}\right)} \end{array}$$

$$\begin{array}{cc} = & \sum_{y_{2}^{n}}P_{Y^{n}|X^{n}}\left(0,y_{2}^{n}|0^{n}\right)\log\frac{Q_{Y^{n}}\left(0,y_{2}^{n}\right)}{Q_{Y^{n}}\left(1,{\bar{y}}_{2}^{k},y_{k+1}^{n}\right)} \end{array}$$

(A24) $$\begin{array}{c} +\sum_{y_{2}^{n}}P_{Y^{n}|X^{n}}\left(1,y_{2}^{n}|0^{n}\right)\log\frac{Q_{Y^{n}}\left(1,y_{2}^{n}\right)}{Q_{Y^{n}}\left(0,{\bar{y}}_{2}^{k},y_{k+1}^{n}\right)} \end{array}$$

$$\begin{array}{cc} = & \sum_{y_{2}^{n}}P_{Y^{n}|X^{n}}\left(0,y_{2}^{n}|0^{n}\right)\log\frac{Q_{Y^{n}}\left(0,y_{2}^{n}\right)}{Q_{Y^{n}}\left(1,{\bar{y}}_{2}^{k},y_{k+1}^{n}\right)} \end{array}$$

(A25) $$\begin{array}{c} +\sum_{y_{2}^{n}}P_{Y^{n}|X^{n}}\left(1,{\bar{y}}_{2}^{k},y_{k+1}^{n}|0^{n}\right)\log\frac{Q_{Y^{n}}\left(1,{\bar{y}}_{2}^{k},y_{k+1}^{n}\right)}{Q_{Y^{n}}\left(0,y_{2}^{n}\right)} \end{array}$$

(A26) $$=\sum_{y_{2}^{n}}\left(\left(1-p\right)P_{Y_{2}^{n}|X_{2}^{n}}\left(y_{2}^{n}|0^{n-1}\right)-pP_{Y_{2}^{n}|X_{2}^{n}}\left({\bar{y}}_{2}^{k},y_{k+1}^{n}|0^{n-1}\right)\right)\log\frac{Q_{Y^{n}}\left(0,y_{2}^{n}\right)}{Q_{Y^{n}}\left(1,{\bar{y}}_{2}^{k},y_{k+1}^{n}\right)}$$

(A27) $$\leq \sum_{y_{2}^{n}}\left|\left(1-p\right)P_{Y_{2}^{n}|X_{2}^{n}}\left(y_{2}^{n}|0^{n-1}\right)-pP_{Y_{2}^{n}|X_{2}^{n}}\left({\bar{y}}_{2}^{k},y_{k+1}^{n}|0^{n-1}\right)\right|k\log\frac{1-p}{p}$$

(A28) $$=\sum_{y_{2}^{k}}\left|\left(1-p\right)P_{Y_{2}^{k}|X_{2}^{k}}\left(y_{2}^{k}|0^{k-1}\right)-pP_{Y_{2}^{k}|X_{2}^{k}}\left({\bar{y}}_{2}^{k}|0^{k-1}\right)\right|k\log\frac{1-p}{p}$$

where (A27) is because of Lemma A1.

Finally,

$$\begin{array}{c} \sum_{y_{2}^{k}}\left|\left(1-p\right)P_{Y_{2}^{k}|X_{2}^{k}}\left(y_{2}^{k}|0^{k-1}\right)-pP_{Y_{2}^{k}|X_{2}^{k}}\left({\bar{y}}_{2}^{k}|0^{k-1}\right)\right| \end{array}$$

(A29) ≤∑i=0k-1k-1i(1-p)pi(1-p)k-1-i-p·pk-1-i(1-p)i

(A30) $$\leq {\left(1-p\right)}^{k}-p^{k}.$$

This concludes the proof.

## Appendix D. Proof of Theorem 6

From the first assumption $\mu_{A}=\gamma_{A}$, we have:

(A31) $$\sum_{y^{n-1}}\mu_{A}\left(y^{n-1}0\right)=\sum_{y^{n-1}}\frac{1}{\left|A\right|}\sum_{x^{n}\in A}P_{Y^{n}|X^{n}}\left(y^{n-1}0|x^{n}\right)$$

(A32) $$=\sum_{y^{n-1}}\frac{1}{\left|A\right|}\sum_{x^{n}\in A_{n0}}P_{Y^{n}|X^{n}}\left(y^{n-1}0|x^{n}\right)+\sum_{y^{n-1}}\frac{1}{\left|A\right|}\sum_{x^{n}\in A_{n1}}P_{Y^{n}|X^{n}}\left(y^{n-1}0|x^{n}\right)$$

(A33) $$=\frac{1-p}{\left|A\right|}\sum_{x^{n}\in A_{n0}}\sum_{y^{n-1}}P_{Y^{n-1}|X^{n-1}}\left(y^{n-1}|x^{n-1}\right)+\frac{p}{\left|A\right|}\sum_{x^{n}\in A_{n1}}\sum_{y^{n-1}}P_{Y^{n-1}|X^{n-1}}\left(y^{n-1}|x^{n-1}\right)$$

(A34) $$=\left(1-p\right)\frac{|A_{n0}|}{\left|A\right|}+p\frac{|A_{n1}|}{\left|A\right|}.$$

Clearly, we can get the following result in a similar manner.

(A35) $$\sum_{y^{n-1}}\mu_{A}\left(y^{n-1}1\right)=p\frac{|A_{n0}|}{\left|A\right|}+\left(1-p\right)\frac{|A_{n1}|}{\left|A\right|}.$$

The ratio of those two is given by:

(A36) $$\frac{\sum_{y^{n-1}}\mu_{A}\left(y^{n-1}1\right)}{\sum_{y^{n-1}}\mu_{A}\left(y^{n-1}0\right)}=\frac{p\frac{|A_{n0}|}{\left|A\right|}+\left(1-p\right)\frac{|A_{n1}|}{\left|A\right|}}{\left(1-p\right)\frac{|A_{n0}|}{\left|A\right|}+p\frac{|A_{n1}|}{\left|A\right|}}.$$

On the other hand, we can also marginalize $\gamma_{A}$:

(A37) $$\sum_{y^{n-1}}\gamma_{A}\left(y^{n-1}0\right)=\frac{1}{s_{A}}\sum_{y^{n-1}}{\left(\prod_{x^{n}\in A}P_{Y^{n}|X^{n}}\left(y^{n-1}0|x^{n}\right)\right)}^{1/|A|}$$

(A38) $$=\frac{1}{s_{A}}\sum_{y^{n-1}}{\left(\prod_{x^{n}\in A_{n0}}\left(1-p\right)P_{Y^{n-1}|X^{n-1}}\left(y^{n-1}|x^{n-1}\right)\prod_{x^{n}\in A_{n1}}pP_{Y^{n-1}|X^{n-1}}\left(y^{n-1}|x^{n-1}\right)\right)}^{1/|A|}$$

(A39) $$=\frac{1}{s_{A}}\sum_{y^{n-1}}{\left({\left(1-p\right)}^{|A_{n0}|}p^{|A_{n1}|}\prod_{x^{n}\in A}P_{Y^{n-1}|X^{n-1}}\left(y^{n-1}|x^{n-1}\right)\right)}^{1/|A|}$$

(A40) $$=\frac{1}{s_{A}}{\left(1-p\right)}^{|A_{n0}\left|/|A\right|}p^{|A_{n1}\left|/|A\right|}\sum_{y^{n-1}}{\left(\prod_{x^{n}\in A}P_{Y^{n-1}|X^{n-1}}\left(y^{n-1}|x^{n-1}\right)\right)}^{1/|A|}.$$

Similarly, we have:

(A41) $$\sum_{y^{n-1}}\gamma_{A}\left(y^{n-1}1\right)=\frac{1}{s_{A}}p^{|A_{n0}\left|/|A\right|}{\left(1-p\right)}^{|A_{n1}\left|/|A\right|}\sum_{y^{n-1}}{\left(\prod_{x^{n}\in A}P_{Y^{n-1}|X^{n-1}}\left(y^{n-1}|x^{n-1}\right)\right)}^{1/|A|}.$$

Thus, the ratio is given by:

(A42) $$\frac{\sum_{y^{n-1}}\gamma_{A}\left(y^{n-1}1\right)}{\sum_{y^{n-1}}\gamma_{A}\left(y^{n-1}0\right)}=\frac{p^{|A_{n0}\left|/|A\right|}{\left(1-p\right)}^{|A_{n1}\left|/|A\right|}}{{\left(1-p\right)}^{|A_{n0}\left|/|A\right|}p^{|A_{n1}\left|/|A\right|}}$$

Since $\mu_{A}=\gamma_{A}$, both ratios should be the same. Let $x=|A_{n0}\left|/|A\right|$, which implies $|A_{n1}\left|/|A\right|=1-x$. Then, we have:

(A43) $$\frac{px+(1-p)(1-x)}{\left(1-p)x+p(1-x\right)}=\frac{p^{x}{\left(1-p\right)}^{1-x}}{{\left(1-p\right)}^{x}p^{1-x}}.$$

If we let $y=\frac{p}{1-p}$, then the above equation can be further simplified to:

(A44) $$\frac{xy+(1-x)}{x+y(1-x)}=y^{2x-1}.$$

**Lemma** **A2.**
*For fixed 0<y<1, the only solutions of the above equation are $x=0,\frac{1}{2},1$.*

**Proof.** It is clear that $x=0,\frac{1}{2},1$ comprise the solution of the following equation.

(A45) $$\frac{xy+(1-x)}{x+y(1-x)}=y^{2x-1}.$$

It is enough to show that:

(A46) $$g_{y}\left(x\right)=\log\left(xy+\left(1-x\right)\right)-\log\left(x+y\left(1-x\right)\right)-\left(2x-1\right)\log y=0$$

can have up to three solutions. Consider the derivative $\frac{\partial }{\partial x}g_{y}\left(x\right)=0$,

(A47) $$\frac{\partial }{\partial x}g_{y}\left(x\right)=\frac{y-1}{xy+1-x}-\frac{1-y}{x+y-xy}-2\log y=0$$

which is equivalent to:

(A48) 2(xy+1-x)(x+y-xy)logy=(1+y)(y-1).

It is a quadratic equation, and therefore, $\frac{\partial }{\partial x}g_{y}\left(x\right)=0$ can have up to two solutions. Thus, $g_{y}\left(x\right)=0$ can have up to three solutions. ☐

This implies that $|A_{n0}|=0,|A|/2$ or |A|. It is clear that the above result holds for all *i* and $A^{c}$, i.e., $|A_{i0}|$ is either 0,|A|/2 or |A|, and $|A_{i0}^{c}|$ is either $0,|A^{c}|/2$ or $|A^{c}|$. These cardinalities should satisfy the following equations:

(A49) $$|A_{i0}\left|+\right|A_{i1}\left|=|A\right|$$

(A50) $$|A_{i0}\left|+\right|A_{i0}^{c}|=2^{n-1}$$

(A51) $$|A_{i0}^{c}\left|+\right|A_{i1}^{c}\left|=\right|A^{c}|$$

(A52) $$|A_{i1}\left|+\right|A_{i1}^{c}|=2^{n-1}$$

for all 1≤i≤n. Since I-compressedness implies $A_{i1}\subseteq A_{i0}$, we have $|A_{i1}\left|\leq \right|A_{i0}|$. Thus, $|A_{i0}|$ should be either |A|/2 or |A| for all *i*. If $|A_{i0}\left|=|A\right|$ for some *i*, then $|A_{i1}|=0$. Since $|A_{i1}^{c}|$ is either $0,|A^{c}|/2$ or $|A^{c}|$, but $A^{c}\neq \emptyset ,\Omega$, we have $|A_{i1}^{c}|=2^{n-1}$. Thus, $A=A_{i0}$ and $\left|A\right|=2^{n-1}$, which implies $A=\Omega_{i0}$.

On the other hand, assume that $|A_{i0}\left|=\right|A_{i1}|=|A|/2$ for all *i*. Since *A* is I-compressed, $x^{i-1}1x_{i+1}^{n}\in A_{i1}$ implies $x^{i-1}0x_{i+1}^{n}\in A_{i0}$. However, we have $|A_{i0}\left|=\right|A_{i1}|$, and therefore:

(A53) $$x^{i-1}1x_{i+1}^{n}\in A_{i1}\Leftrightarrow x^{i-1}0x_{i+1}^{n}\in A_{i0}$$

and equivalently,

(A54) $$x^{i-1}1x_{i+1}^{n}\in A\Leftrightarrow x^{i-1}0x_{i+1}^{n}\in A$$

for all *i*. It can only be true when A=∅ or Ω, which contradicts our original assumption. This concludes the proof.
